# The effect of trends in health and longevity on health services use by older adults

**DOI:** 10.1186/s12913-015-1239-8

**Published:** 2015-12-24

**Authors:** Bram Wouterse, Martijn Huisman, Bert R. Meijboom, Dorly J.H. Deeg, Johan J. Polder

**Affiliations:** Tranzo Scientific Center for Care and Welfare, Tilburg School of Social and Behavioral Sciences, Tilburg University, Tilburg, The Netherlands; Center for Public Health Forecasting, National Institute for Public Health and the Environment, Bilthoven, >The Netherlands; CPB Netherlands Bureau for Economic Policy Analysis, P.O. Box 80510, The Hague, 2508 GM >The Netherlands; EMGO + Institute on Health and Care Research, Department of Epidemiology & Biostatistics, VU University Medical Center, Amsterdam, >The Netherlands; Department of Sociology, VU University, Amsterdam, >The Netherlands; Department of Psychiatry, VU University Medical Center, Amsterdam, >The Netherlands

**Keywords:** Health expenditures, Scenario analysis, Aging, Health trends

## Abstract

**Background:**

The effect of population aging on future health services use depends on the relationship between longevity gains and health. Whether further gains in life expectancy will be paired by improvements in health is uncertain. We therefore analyze the effect of population ageing on health services use under different health scenarios. We focus on the possibly diverging trends between different dimensions of health and their effect on health services use.

**Methods:**

Using longitudinal data on health and health services use, a latent Markov model has been estimated that includes different dimensions of health. We use this model to perform a simulation study and analyze the health dynamics that drive the effect of population aging. We simulate three health scenarios on the relationship between longevity and health (expansion of morbidity, compression of morbidity, and the dynamic equilibrium scenario). We use the scenarios to predict costs of health services use in the Netherlands between 2010 and 2050.

**Results:**

Hospital use is predicted to decline after 2040, whereas long-term care will continue to rise up to 2050. Considerable differences in expenditure growth rates between scenarios with the same life expectancy but different trends in health are found. Compression of morbidity generally leads to the lowest growth. The effect of additional life expectancy gains *within* the same health scenario is relatively small for hospital care, but considerable for long-term care.

**Conclusions:**

By comparing different health scenarios resulting in the same life expectancy, we show that health improvements do contain costs when they decrease morbidity but not mortality. This suggests that investing in healthy aging can contribute to containing health expenditure growth.

## Background

The influence of population aging on health services use depends on the relationship between longevity and health. Whether increases in life expectancy result in higher health care costs depends on whether additional life years are spent in good or poor health. Moreover, use of different types of health services, such as hospital care or long-term care (LTC), will be related to particular aspects of health. However, most studies that project future health spending either assume that the relationship between mortality and health is fixed, or consider only one dimension of health. These studies thus ignore the importance of the potentially diverging trends between mortality and health, and between different dimensions of health for the growth of health spending. In this paper, we use scenario analysis to assess how the influence of longevity gains on health expenditure growth is driven by the uncertain relationship between longevity and health.

Empirical studies show diverging trends between different dimensions of health. Most Western countries have been experiencing longevity gains over the past decades [1]. At the same time, the number of years spent with chronic diseases seems to have been increasing [2]. In contrast, life years spent with disability seem to be declining or stable [3]. In general, the relationship between further increasing life expectancy and trends in health seems to depend strongly on the dimension of health under consideration. Also, trends in health are surrounded by considerable uncertainty: findings differ between countries and studies, especially for disability [3, 4].

Despite the uncertainty regarding the relationship between mortality and health, a substantial part of the research on cost of aging treats this relationship as fixed. In particular, the time to death literature uses proximity to death as a measure for morbidity [5, 6]. This literature implicitly assumes that trends in health and mortality coincide. In that case, longevity gains do not lead to increases in health expenditures but merely to postponement of costs to later ages. These type of studies thus ignore the possibility of longevity gains without improvement in underlying health, which would most likely lead to higher health spending.

Other studies do differentiate between mortality and health, but focus on a single health indicator. A number of studies include disability trends in predictions of health services use and expenditures in the U.S. [7–9]. A similar study exists for the Netherlands [10]. Since trends in dimensions of health diverge, the use of a single health indicator does not suffice to capture all relevant dynamics. This is especially relevant because different health dimensions relate to different types of health services. For instance, use of hospital care is strongly related to chronic diseases, whereas LTC use is mostly related to disability [11].

Goldman et al. do use a more diverse set of health variables in their projections for the U.S. [12]. However, they only consider scenarios in which improvements in life expectancy are caused by improvements in health. Again, the not unlikely scenario that life expectancy changes can occur without an improvement of health is not taken into account. The resulting differences in health expenditures between their scenarios are therefore small. Policy-oriented studies, such as those by the European Commission and the OECD [13, 14] do consider to what extent different health scenarios influence the effect of life expectancy growth on health spending. These studies generally lack explicit modelling of the underlying relationship between longevity and health.

In this study we directly model this relationship. The model is based on a latent health variable that incorporates a large set of health indicators. In contrast to other studies, we do not treat longevity as an outcome of health improvements but instead consider different health scenarios resulting in the same increase in life expectancy. We relate the scenarios to the three most common hypotheses on the relationship between mortality and health and to the empirical evidence on trends in health. Our aim is to gain insight into the health dynamics that drive the effect of aging on expenditure growth. We apply the model to health services projections in the Netherlands between 2010 and 2050. We focus on hospital care, home care and institutional LTC.

### Trends in health

The three most common hypotheses on the relationship between longevity and health have been formulated in the 1980s [15]. In the expansion of mortality hypothesis, medical progress is expected to lead to an increasing survival of people in poor health [16, 17]. As a result, the number of years spent in poor health expands. Instead, the compression of morbidity hypothesis expects that medical progress is mostly aimed at improvements in health, resulting in fewer years spent in poor health [18]. Finally, the dynamic equilibrium hypothesis assumes a tradeoff between increasing prevalence and decreasing severity of chronic diseases, resulting in a constant proportion of life spent in poor health [19].

In most Western countries, life expectancy has increased by about 30 years during the last century [4]. Almost all studies foresee a further increase of life expectancy in the coming decades, e.g. [1]. In the Netherlands, life expectancy at birth was 78.8 for men and 82.7 for women in 2010. A further, almost linear, increase is predicted, resulting in a life expectancy at birth of 83.8 for men and 88.1 for women by 2050 [20]. The forecast for remaining life expectancy at 65 is 21.1 years for men and 24.6 years for women, compared to 16.7 and 20.1 years, respectively, in 2006 [20].

The relationship between health and longevity depends on the dimension of health under consideration. Here, we discuss evidence on trends in chronic diseases, disability, and lifestyle. The prevalence of chronic diseases is rising in most countries, because the survival of people with one or more chronic diseases is growing [2, 4, 21]. The rising trend in chronic diseases might partly be the result of improved medical treatments of some fatal conditions that do not change the age-specific onset of those conditions. In the Netherlands, a decline in life expectancy without chronic diseases from 53 to 48 years for men and 52 to 43 years for women between 1983 and 2007 due to an increase in chronic conditions has been observed [22, 23].

Evidence on trends in functioning and disability is mixed, sometimes even between different studies in the same country. In the U.S., a number of studies have found improvements in functioning and a decline of disability in the 1980s and 1990s [3]. A study for twelve OECD countries, specifically on severe disability, finds that five countries, including the Netherlands, show a decline in severe disability, but a same number of countries show an increasing trend [24]. Another study reports evidence for several countries that the expected number of years spent with severe disability is declining or stable, while the number of years spent with moderate disability is increasing [21]. This trend is also observed for the Netherlands [25]. A meta study using results from five Dutch surveys also finds evidence for this trend, but shows mixed findings between surveys [26]. It seems that severe disability is strongly linked to the last phase of life, whereas mild disability is not [27–29], implying that an expansion of life expectancy increases life years spent with mild disability, but years spent with severe disability remain stable.

Lifestyle differences between cohorts show diverging trends. Obesity rates in the U.S. have been increasing for successively born cohorts, while trends in smoking behavior are optimistic [3]. An important positive influence is the rise in education level [3, 21]. In the Netherlands, an increase in the prevalence of obesity and a slight decline in smoking, especially for men, have also been observed [22]. Higher prevalence of obesity, higher alcohol consumption and lower physical activity of 55 to 64-years old in 2002–2003 compared to 1992–1993 have been found in the Netherlands [30].

Empirical findings do not decisively show which hypothesis is right. Trends differ between health dimensions. The findings on the trends in disability are mixed. It is also unclear how the diverging trends in the lifestyle of younger cohorts will affect the prevalence of chronic diseases and disability in the future. To account for the uncertainty surrounding the relationship between mortality and heath, we base our simulation study on all three hypotheses.

## Methods

Our aim is to make scenarios of future health and health service use, including trends in different dimensions of health. The challenge is to capture all relevant relationships between different aspects of health, while at the same time keeping the model parsimonious enough to be used in a simulation exercise. For this purpose, we use a latent Markov model that we have developed earlier [31]. In this model, the relationship between a set of health indicators and health services is modeled through a single discrete variable with four states (including death). Annual transition probabilities between the states of this latent health variable are also estimated. In order to obtain projections of all relevant dimensions of health, only simulations of the latent variable have to be made. We first discuss how the Latent Markov model is estimated, and then show how we use this model to simulate different health scenarios.

### Data

The model is estimated on a combined dataset of longitudinal health survey data linked with registry data on health services use. The health survey we use is the Longitudinal Ageing Study Amsterdam (LASA) [32]. This is a study among older adults in the Netherlands. The study started in 1992 with 3107 respondents and a new cohort was added in 2002. We link this survey to registry data on hospital use between 1995 and 2007, and long term care use between 2004 and 2007. From this data we create an estimation sample for the years 1995–2007. The sample is described more extensively in Appendix A.2.

Seven indicators of health and disability are used in the model, covering physical as well as mental aspects of health. Self-perceived health is measured on a five point scale. Physical functioning is measured by a self-reported indicator as well as an objective indicator. The first indicator consists of three items, each pertaining to a mobility activity in daily life. The indicator is a total score, ranging between 1 (no limitations) and 4 (limitations for all activities) [33]. The second indicator is a performance test, measuring the time it takes for the respondent to put on and take off a cardigan. Limitations in daily activities are measured with the Global Activity Limitation Indicator (GALI) [34]. The presence of chronic diseases is also self-reported. The mental aspects of health included in the study are depressive symptoms and cognitive impairments. For depressive symptoms the Center for Epidemiological Studies Depression Scale (CES-D) is used [35]. Cognitive impairments are measured by the Mini Mental States Examination (MMSE) [36].

### The latent Markov model

We use a Latent Markov Model to describe the relationship between health and health services use over time. The model assumes that an individual’s health and his health services use are determined by an underlying unobserved health variable. The model estimates this latent variable based on the joint distribution of the separate health indicators as well as the distribution of health services use. The latent variable has a fixed number of states. Changes in latent health over the life course are modeled through annual transitions between the states. These transitions are modeled as a Markov process. Estimation of the model is described in Appendix A.1, and in more detail in [31].

**Fig. 1:**
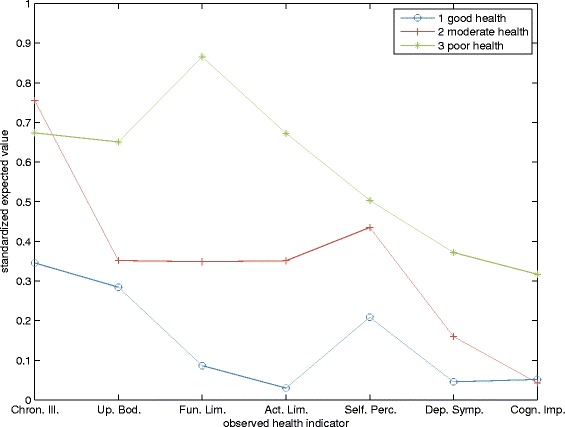
The relationship between the latent health variable and the observed health indicators. Expected values of the observed health indicators for each latent health state. Values have been standardized to lie between 0 (best outcome) and 1 (worst outcome)

In the specification of the model that we use for the simulations, the latent variable has four states: three health states and death. An individual’s latent health state determines how likely he is to report a particular health outcome (as measured by the seven indicators) as well as his likelihood of health services use. For instance, someone in good latent health will have a higher probability of reporting good self-perceived health than someone in poor latent health. Figure 1 shows how the states of the latent variable are related to the observed health indicators. The figure shows the expected outcomes, which for ease of comparison have been standardized to lie between 0 (best outcome) and 1 (worst outcome). State 1 is related to good health for all indicators. State 2 is related to moderate health: a high probability of having chronic diseases, moderate probability of having disability, moderate self-perceived health, and low probabilities of having cognitive impairments or depression. State 3 is poor health with a high probability of disability, cognitive impairments, and depression. The probability of reporting poor self-perceived health in state 3 is only slightly higher than in state 2.

**Fig. 2:**
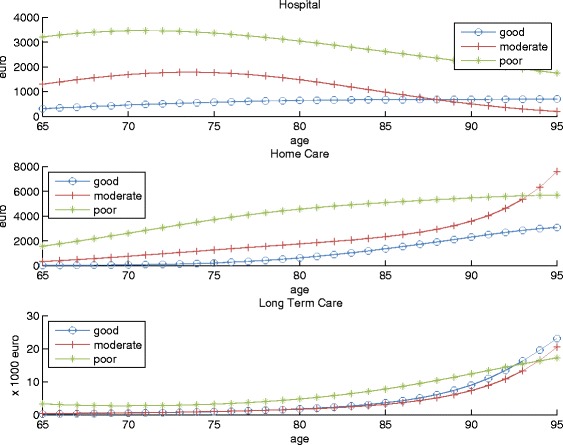
The relationship between the latent health variable and costs of health services use. The age curve of expected costs of health services use (hospital care, home care, long term care) for men (with baseline characteristics) for each latent health state

Figure 2 shows how the states of the latent variable are related to costs of health services use. The figure shows the expected costs of the three different types of health services for men by age. All other covariates are set at baseline levels. Up to age 85–90, good health is related to lowest expected costs for all three types of services, and poor health is related to highest costs. Differences in expected costs between good and moderate health are larger for hospital and home care use than for LTC. At the highest ages, the poor health state is not always associated with the highest expected costs. For instance, at ages higher than 93, expected costs of home care use are higher for men in moderate health than men in poor health. These differences are related to substitution between types of care or mortality differences between states.

### Scenario analysis

Using the latent Markov model, we can perform cohort simulations for the period 2010–2050. We start with the Dutch older population between 65 and 95 in base year 2010. The initial distribution over the health states of this population is based on the age- and sex-specific distribution of health states in the LASA sample. Based on the transition probabilities from the model, the health and expenditure distribution for the 2010 cohorts can be calculated consecutively for the following years. Each year, a new cohort of 65-years old men and women is added. The size of these new cohorts is obtained from population projections of Statistics Netherlands [37].

There are two ways in which different health scenarios can be implemented in the simulation model: First, we can change the proportion of people in a particular health state (initial health profile), for new cohorts of 65-years old. Such a change can be seen as a consequence of health changes that materialize at younger ages, before 65. Second, we can change the transition probabilities between health states in the model. These changes are related to effects of trends in heath that materialize after 65. The idea of the scenario analysis is that we start with a given life expectancy in 2050, and then consider different health scenarios to get there. For example, a rise in life expectancy can be achieved by lowering the age-specific probabilities of transiting to poor health in favor of remaining in good health. Instead, the same rise can be achieved by lowering the probability of dying from poor health in favor of remaining in poor health.

The implementation of each scenario starts with the given remaining life expectancy in 2050. For each health scenario we select the transition probabilities that have to be adjusted according to the scenario. In general, we adjust these probabilities by a single proportional change each year. For instance, we can choose to reduce the probability of dying (from all other states) by *x* % per year. We can assume that the additional surviving individuals remain in their current health state. Because we use a single adjustment parameter *x*, the value of this parameter can be determined numerically based on the desired life expectancy in 2050. In some scenarios we change both the initial health distribution at 65 and the transition matrix. In those cases, we first set the change in the initial health distribution to a level we consider adequate, and then calculate the required change in transition rates.

#### Implementation

We set remaining life expectancy at 65 in 2050 at two given values: the forecasted values of 21.1 for men and 24.6 for women [20], and the more optimistic values of 24.1 for men and 27.6 for women. By using two values we can assess the effect of changes in life expectancy growth as well as the influence of health on growth of health services use.

We use three overarching scenarios, based on the morbidity hypotheses described earlier. We divide these into several sub scenarios. In the first scenario, we let the life expectancy gain be the result of an overall decrease in mortality rates. This scenario is in accordance with the expansion of morbidity hypothesis. Changes in lifestyle do not lead to an improvement in age specific health, and medical technology is mostly applied to decrease mortality for individuals in poor health without changing the age specific onset of diseases. We implement this scenario by decreasing the probability of dying in each health state by the same proportion. As a result, the number of years spent in poor health increases.

The second scenario relates to the compression of morbidity hypothesis. Prevention and changes in lifestyle lead to better population health. As a result, life expectancy gains are paired with longer remaining lifetime spent in good health. We implement this scenario by increasing the probabilities of remaining in good or moderate health, compared to worse health outcomes. In the third scenario, we adhere to the dynamic equilibrium hypothesis: life years spent with one or more chronic diseases increase, whereas the number of life years spent with severe disability remains constant. The remaining lifetime spent in moderate health, associated with chronic diseases but only mild disability, is prolonged. The time spent in poor health, associated with severe disability, is kept constant. We implement this scenario by decreasing the probabilities of going from good or moderate health to poor health and death in favor of going to or remaining in moderate health. To be able to set both the remaining lifetime as well as the time spent in poor health, we also decrease the probability of dying in poor health with a separate growth rate.

For the first scenario, we keep the initial health profile at 65 at its 2010 level. The scenario can thus be modeled by only changing the transition probabilities. For the other two scenarios, we model health changes either solely through changes in the transition probabilities, or by a combination of changes in the initial health profile and changes in the transition probabilities. In the combined specification of the compression scenario, we let the proportion of individuals in good health at 65 gradually go to 100 % in 2050. Remaining annual decrease in transition probabilities needed for the desired life expectancy can then be numerically determined. In the combined specification of the dynamic equilibrium scenario, we let the initial proportion of 65-years old in moderate health increase to 45 % in 2050.

We end up with eight sub scenarios: a modest and extreme life expectancy version for each scenario, and for the compression and dynamic equilibrium scenarios an additional version of the modest life expectancy scenario, where only transition probabilities are changed. Additionally, we include a baseline scenario in which the initial health profile and transition probabilities are kept at their 2010 values.

As an illustration of how the scenarios are implemented, Table 1 shows the distribution of initial population health at 65, and the transition matrix for men at 75, in 2050 in the different scenarios. Scenarios with only a change in transition probabilities are indicated by an o, scenarios with changes in the initial health state by +, and extreme life expectancy scenarios by ++.

**Table 1:** Initial population health at 65 and transition matrix at 75 for men in 2050, under different health scenarios

Population health for men at 65				
	Share in 2050			
Scenario	Good	Moderate	Poor	Deceased
Baseline (1o, 1 ++, 2o, 3o)	0.66	0.27	0.06	0.00
Compression (2 +, 2 ++)	0.99	0.01	0.00	0.00
Dynamic (3 +, 3 ++)	0.51	0.44	0.05	0.00
Transition matrix for men at 75 in 2050				
State at *t*	State at *t*+1			
	Good	Moderate	Poor	Deceased
Baseline				
Good	0.87	0.07	0.04	0.02
Moderate	0.00	0.87	0.10	0.03
Poor	0.00	0.04	0.81	0.15
1o Expansion of morbidity				
Good	0.92	0.04	0.02	0.01
Poor	0.00	0.04	0.88	0.09
1 ++ Expansion of morbidity, high life exp.				
Moderate	0.00	0.95	0.04	0.01
Poor	0.00	0.04	0.90	0.06
2o Compression of morbidity. Transition				
Good	0.94	0.03	0.02	0.01
Moderate	0.00	0.94	0.05	0.01
Poor	0.00	0.04	0.81	0.15
2 + Compression of morbidity. Transition and initial				
Good	0.93	0.04	0.02	0.01
Moderate	0.00	0.93	0.06	0.02
Poor	0.00	0.04	0.81	0.15
2 ++ Compression of morbidity, high life exp. Transition and initial				
Good	0.96	0.02	0.01	0.01
Moderate	0.00	0.96	0.03	0.01
Poor	0.00	0.04	0.81	0.15
3o Dynamic equilibrium. Transition				
Good	0.87	0.10	0.02	0.01
Moderate	0.00	0.94	0.05	0.01
Poor	0.00	0.04	0.84	0.12
3 + Dynamic equilibrium. Transition and initial				
Good	0.87	0.10	0.02	0.01
Moderate	0.00	0.94	0.04	0.01
Poor	0.00	0.04	0.84	0.12
3 ++ Dynamic equilibrium, high life exp. Transition and initial				
Good	0.87	0.11	0.01	0.01
Moderate	0.00	0.97	0.03	0.01
Poor	0.00	0.04	0.86	0.11

We present the effects of the different scenarios from three perspectives. First, we take an individual perspective, and consider the differences in remaining life expectancy and costs of health services use over remaining lifetime at 65 in 2050. Second, we describe the cross sectional age profile of population health. Third, we look at the growth in aggregated health services use and expenditures between 2010 and 2050.

## Results

**Fig. 3:**
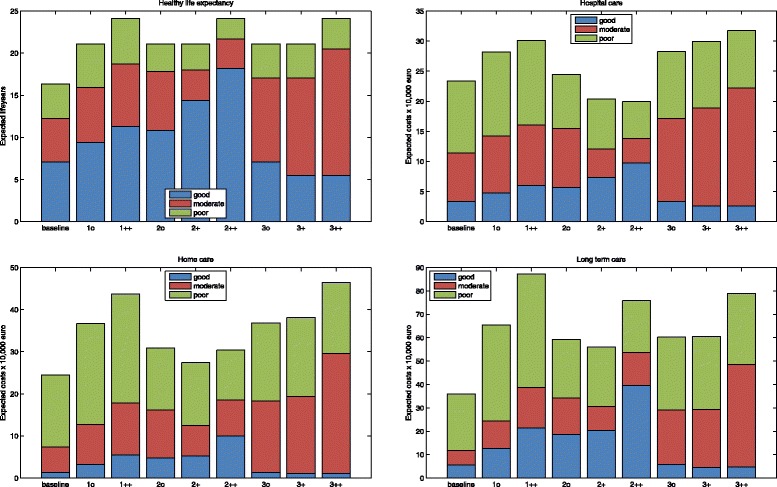
The expected remaining lifeyears and health expenditures spent in each health state for 65-years old for men under different scenarios. 1 expansion of morbidity, 2 compression of morbidity, 3 dynamic equilibrium. o indicates the scenarios without changes in the health of new cohorts of 65-years old, + the scenarios including health changes at 65, and ++ scenarios with additional gains in life expectancy

Remaining healthy life expectancies at 65 for men in 2050 are depicted in Fig. 3. In the baseline scenario, healthy life expectancy is equal to that in 2010. In the other scenarios, life expectancy is either 21.1 or 24.1. In the extension of morbidity scenarios the proportion of remaining life spent in each state is equal to the baseline scenario. As a result, the *absolute number* of years spent in poor health increases. The compression of morbidity scenarios show an increase in the number of years spent in good and moderate health. The compression scenario that includes changes in initial health at 65 results in more number of years spent in good health than the scenario that only includes health changes after 65. In the dynamic equilibriums scenarios time spent in moderate health, with chronic disease but only mild disability, increases. Time spent in poor health remains constant. In the scenario without changes in initial health, the time spent in good health is equal to the baseline scenario. In the scenario including changes at 65 the time spent in good health is shorter, due to the increasing proportion of 65-years old in moderate health.

Figure 3 also shows the individual expected costs of health services use in each health state for men at 65 over remaining life in 2050, for hospital care, home care, and institutional LTC separately. Remaining lifetime expenditures show some considerable differences between scenarios, especially between the compression of morbidity scenarios and the others. Hospital expenditures are 23,000 Euros in the baseline scenario. Hospital expenditures are 5000 Euros higher in expansion of morbidity scenario and 7000 Euros higher in the extreme life expectancy variant of this scenario. In the improvement of health scenarios, hospital expenditures are slightly higher than in the baseline scenario for the moderate life expectancy variant without initial health changes, whereas expenditures are lower in the other two variants (about 20,000 Euros). Hospital expenditures range from 29,000 Euros to 32,000 Euros in the dynamic equilibrium scenario.

Home care expenditures over remaining lifetime are 24,500 Euros in the baseline scenarios. Expenditures are higher in all other scenarios. Of the other scenarios, expenditures are lowest in the different versions of the compression of morbidity scenario (27,000–30,000 Euros), and range from 36,000–46,000 Euros in the other scenarios. Institutional LTC expenditures are again lowest in the baseline scenario (36,000 Euros). In the other scenarios, expenditures range from 55,000 Euros in the compression scenario without initial health changes to 86,500 Euros in the extreme life expectancy variant of the expansion scenario. In contrast to hospital and home care, the extreme versions of all scenarios result in higher lifetime LTC expenditures compared to other versions of the same scenario.

**Fig. 4:**
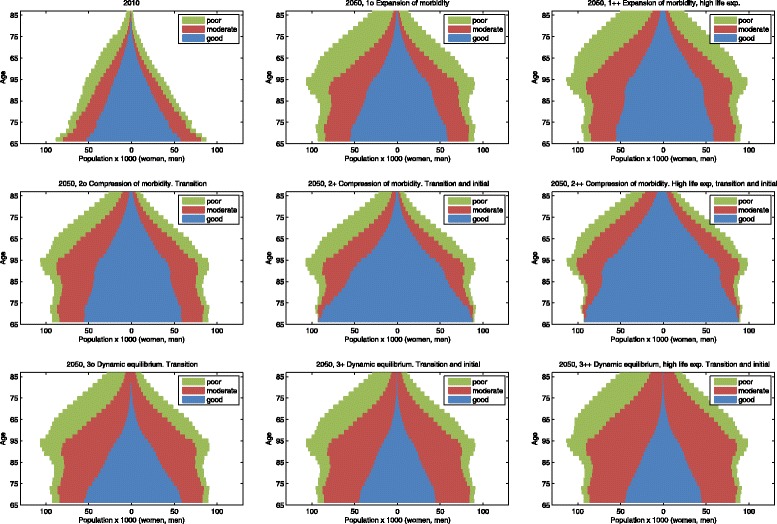
Health composition of the population in 2010 and projection for 2050 under different scenarios. The number of people in each health state per age group (women left, men right)

Figure 4 shows the health composition of the older population in 2010, and in 2050 for each scenario. All scenarios show the well-known baby boom effect: the broadest part of the pyramid moves upward over time. The population pyramids in the expansion of morbidity scenarios show the same form as in 2010, only broader. The compression of morbidity scenarios show an increase of the proportion of individuals in good health, mainly at the younger ages at the bottom of the pyramid. The dynamic equilibrium scenarios show an increase of the number of people in moderate health (without severe disability), especially at the middle and top of the pyramid. When we compare the scenarios where only the transition probabilities are changed to the scenarios where the initial age profile is also changed, we can see that in the latter a larger part of the health changes occurs at the bottom of the population pyramid. The scenarios with the more extreme raise in life expectancy have a larger number of people at the top of the population pyramid.

**Fig. 5:**
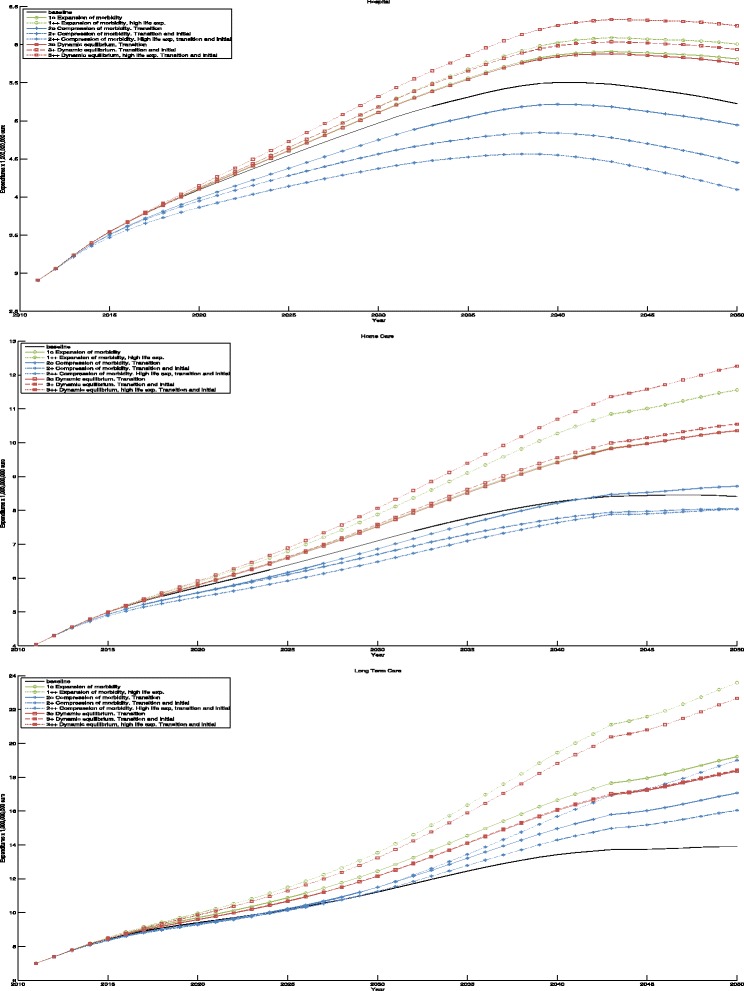
Predictions of expenditures between 2010 and 2050, for hospital care, home care, and long-term care, for each scenario

The projections of health care expenditures over the years 2010–2050 are depicted in Fig. 5. Of the scenarios with the more conservative life expectancy prediction, the dynamic equilibrium scenario including initial health changes and the expansion of morbidity scenario result in the highest hospital expenditures. The lower prevalence of disability in the dynamic equilibrium scenario thus seems to matter little for hospital expenditures. The compression of morbidity scenarios, where the prevalence of chronic diseases is lower, result in considerably lower hospital expenditures. The large difference between the compression scenario without improvement in initial health at 65 and the same scenario with improvement in initial health is also noteworthy. Hospital expenditures in the extreme life expectancy variants of the expansion and dynamic equilibrium scenarios are slightly above spending in the standard variants. In contrast, hospital spending in the compression of morbidity scenario with higher life expectancy is lower than in the other versions of this scenario.

Whereas hospital expenditures decrease or stabilize after 2040, home care and institutional LTC expenditures rise over the whole time interval in all scenarios, except the baseline. Home care expenditures are highest in the dynamic equilibrium scenario with extreme life expectancy, followed by the moderate life expectancy variant of the expansion scenario and the dynamic equilibrium scenario without changes in initial health. The moderate life expectancy variants of the compression of morbidity scenarios are related to lowest expenditures. However, the scenario without changes in initial health surpasses the baseline scenario around 2043. Again, additional life expectancy gains lead to higher expenditures in the expansion and dynamic equilibrium scenario.

Institutional LTC expenditures are highest in the moderate life expectancy variant of the expansion of morbidity scenario. The moderate life expectancy variants of the dynamic equilibrium scenario are second and third, but the difference with the expansion scenario is relatively larger than for hospital and home care. Here, the lower prevalence of disability in the dynamic equilibrium scenarios compared to the expansion of morbidity scenarios does have a strong effect on expenditures. Although expenditures in compression of morbidity scenarios are lowest, the difference with the other scenarios is smaller than for hospital care. For institutional LTC, additional life expectancy gains have a relatively large impact on spending in the expansion and dynamic equilibriums scenarios. LTC expenditures in the extreme life expectancy version of the compression of morbidity scenario are lower than the other variants of this scenario, but they seem likely to surpass these scenarios after 2050. Not including the baseline scenario, the annual growth rates of expenditures range from 0.88 to 1.98 for hospital care, 1.78 to 2.89 for home care, and 2.15 to 3.16 for institutional LTC.

## Discussion

We have analyzed the consequences of population aging based on three health scenarios: an expansion of morbidity, a compression of morbidity, and a dynamic equilibrium. The way the scenarios are implemented is extreme: in each scenario, adjustment of the selected health parameters explains the total change in life expectancy. In reality, it might be more likely that life expectancy gains are a result of a mix of scenarios. Extreme scenarios have the advantage of showing the boundaries of the effect of health on health expenditures. We find that the compression of morbidity scenarios lead to the smallest growth in expenditures. The dynamic equilibrium scenarios result in equal or higher expenditures for hospital and home care services as the expansion of morbidity scenarios, but LTC spending is lower.

In contrast to other studies [12, 38], we find substantial differences in lifetime expenditures between health scenarios. This difference can be explained by the fact that in these studies improvement of health always leads to longer life. For scenarios where we have also assumed a life extending effect of better health we find similar results. In the extreme life expectancy version of the compression of morbidity scenario we assume that the additional gain in longevity is the result of a further improvement of health. This indeed provides the same tradeoff found in earlier studies: lower costs per life year are offset by an increase in the number of life years. The extreme life expectancy version of the compression scenario results in some savings in lifetime hospital expenditures compared to the other versions, but these are compensated by an increase in expected LTC use at older ages. Comparison *between* health scenarios resulting in the same life expectancy show that health improvements do contain costs when they decrease morbidity but not mortality. Grootjans- van Kampen et al. [39] come to a similar conclusion in a study focusing on differences between diseases: prevention of lethal diseases tends to increase health care costs, while prevention of non-lethal diseases can lead to savings.

The real growth rate of aggregated health care expenditures in the Netherlands during the last decade has been around 4.4 percent [40]. Our results confirm earlier findings that aging and underlying health changes can only explain a part of health care expenditure growth. The growth rates for LTC and home care are somewhat higher than reported in another Dutch study [10]. This difference can be attributed to the fact that we study a longer time period. LTC volume projections by the Netherlands Institute for Social Research [41] for 2010 to 2030 are within the range of our scenario projections over the same period.

The time trends in aggregated expenditures show substantial differences between health scenarios. Again, differences are generally larger between health scenarios with the same life expectancy than between versions of the same health scenarios with different life expectancy. Our findings, to some extent, support the conclusion of the time to death literature that longevity gains do not necessarily lead to expenditure increases. Such result is in line with the finding that increasing life expectancy in the U.S. by gradually moving American cohorts to the better health status enjoyed by Western Europeans could lead to substantial health care savings [42]. However, as we have shown, cost containment can only be achieved when increasing life expectancy is indeed a result of health improvements.

An important limitation of our study is that we do not consider other drivers of health expenditure growth besides demographics and health. The focus of our research is on the way in which different interactions between morbidity and mortality affect health spending, and not on identifying all relevant drivers of health spending. Researchers who want to include other drivers, such as the development of new medical technologies, should be aware of interaction effects. For instance, it seems that new medical technologies lead to a stronger rise in health spending for people in poor health than it does for people in better health [43].

Three recommendations for policy makers can be drawn from this study. First, based on the empirical literature we cannot expect a decrease in the prevalence of chronic diseases among the older population in the short run. In this respect, the more optimistic expenditure development of the compression scenario seems unlikely to be realized. However, investing in lifestyle changes at younger ages can pay off in the long run through a decrease in chronic diseases among the *future* old. In fact, comparison between the compression scenarios with and without changes in health before 65 shows that life expectancy gains due to health improvements at younger ages lead to less expenditure growth than when these same gains are solely due to health improvements after 65.

Second, the fact that LTC costs are lower in the dynamic equilibrium scenario than in the expansion of morbidity scenario shows the potential for cost containment through preventing disability. Given that prevention of chronic diseases among older adults can be difficult in the short term, policies focusing on the improvement of independence of older adults with chronic disease could contain LTC costs. Third, in all scenarios population aging leads to a rise in health services use. Health improvement policies thus have an important but also slightly limited role in containing health care costs.

## Conclusions

The effect of population aging, and especially longer life, on health services use depends on trends in diverse aspects of underlying health. In this study, we have performed a scenario analysis based on three common hypotheses about this relationship, using a combined measure of health. We find substantial differences in health expenditure growth between the most optimistic and the most pessimistic health scenarios. By comparing different health scenarios resulting in the same life expectancy, we have shown that health improvements do contain costs when they decrease morbidity but not mortality. Scenarios in which the prevalence of chronic diseases increases, but the disabling effect of these diseases decreases (dynamic equilibrium) lead to relatively high growth in hospital services use, but a relatively limited growth in LTC use. The results suggest that investing in healthy aging can contribute to containing health expenditure growth.

## Endnote

^1^ The sample is randomly selected from municipal records, and representative of the Dutch population initially aged 55–85 years in terms of socio-geographic distribution and distribution of population density. The representativeness is maintained because the attrition is largely due to mortality, which also occurs in the general population.

## Appendix A: Methods and data

### A.1 Specification of the latent Markov model

The model that we use for our simulations is a Latent Markov model. The model is based on an unobserved (latent) discrete health variable that determines the probability distribution of a number of observed health indicators as well as health expenditures over time. The model consists of two components. First, a measurement component defining the relationship between the value of the latent variable and the observed health indicators and health care expenditures at a particular time. Second, a structural component, modeling individual changes in the latent variable over time [44]. Let *η*_*i,t*_ be the value of latent health variable *η* for individual *i* in year *t*, where *η* is a discrete variable with *M* number of states. The first part of the model describes how the values of *J* different observed discrete health indicators $y^{1}_{\textit {i,t}},\ldots,y^{\,J}_{\textit {i,t}}$, and health care costs *c*_*i,t*_ depend on the value of *η*_*i,t*_. For each state *m* of *η* we define a separate conditional probability distribution for each health indicator *j*, $P\left (y^{\,j}_{\textit {i,t}}=k|\eta _{\textit {i,t}} = m\right)$, and a probability density function for expenditures *f*(*c*_*i,t*_|*η*_*i,t*_=*m, x*_*i,t*_).

For the functional form of the conditional probabilities of the observed health indicators we use a multinomial logit specification. For the conditional density functions of the costs we use a two-part model specification. The first part is a logit, describing the probability that individual *i* uses any health care in year *t*. The second part is a GLM with a Gamma distribution, describing the distribution of health care costs for individual *i*, conditional on the fact that individual *i* uses health care in year *t*. The logits for the observed health indicators do not include covariates. The two-part models for the costs include *sex*, *partnerstatus*, *age*, *educationlevel*, and calendar year dummies as covariates. Thus, the relationship between the latent variable and the distribution of health care costs depends on these covariates.

The structural part of the model describes how the latent health variable changes over time. For this purpose we use a first-order Markov model with annual transitions between states. The Markov assumption entails that the state of *η* in year *t* only depends on the state of *η* in year *t*−1. We model *P*(*η*_*i,t*_=*m*|*η*_*i,t*−1_=*l*;*x*_*i,t*_) using a separate multinomial logit for each state *l*∈*M* of *η*_*i,t*−1_. We include the same set of covariates as in the conditional cost model except calendar year.

To estimate the model we have to choose the number of states *M* of the latent variable. Given *M*, we can jointly estimate both parts of the model by maximizing the likelihood of the observed combinations of health indicators and health care costs over all periods of time *t*=1,..,*T* and all individuals *i*,=1,..,*N*. Let’s define *θ* as a vector containing all parameters of the multinomial logits and two-part models that have to be estimated. Then, the likelihood for an individual *i* is

(1) $$  \begin{aligned} P&\left(c_{i},{y^{1}_{i}},\ldots,{y^{J}_{i}}|x_{i},\theta\right)\\ &= \sum_{m_{0}=1}^{M} \sum_{m_{1}=1}^{M} \!\!\ldots \!\!\sum_{m_{T}=1}^{M} P(\eta_{i,0}=m_{0}|\theta) \prod_{t=1}^{T} P(\eta_{i,t}=m_{t}|\eta_{i,t-1}\\ &\quad= m_{t-1};x_{i,t-1};\theta) \prod_{t=0}^{T} g_{m_{t}}(c_{i,t},y_{i,t}|x_{i,t};\theta), \end{aligned}  $$

where $g_{m}\,\left (c_{\textit {i,t}},y_{\textit {i,t}}|x_{\textit {i,t}}\right)\!\!\!\!\!\!\!\,=\,\!\!\!\!\!\!f\left (c_{\textit {i,t}}|x_{\textit {i,t}},\eta _{\textit {i,t}}=m\right) \,\prod _{\,j\,\,\,=\,\,\,1}^{\,J} P \left (y^{\,j}_{\textit {i,t}}|\eta _{\textit {i,t}}=m\right)$ is the measurement component, and *P*(*η*_*i*,0_=*m*_0_|*θ*) is the initial state probability. The log-likelihood over all individuals can be maximized using the expectation maximization algorithm. The number of states of the latent variable can be determined by comparing model fit between specifications. Death is defined as a separate state.

### A.2 Data

The Latent Markov model is estimated on Dutch data on health and hospital expenditures over the period 1995–2007. Health indicators are obtained from the Longitudinal Aging Study Amsterdam (LASA) and hospital use is based on register data. The relationship between the latent variable and LTC and home care costs is estimated ex-post. For this we use another register available for the years 2004–2007. The different datasets are combined through (anonymized) linkage to the Dutch Municipal Register (GBA) which contains basic information on everyone enlisted in a Dutch municipality.

The Longitudinal Ageing Study Amsterdam (LASA) is an ongoing observational study on predictors and consequences of changes in emotional, physical, cognitive and social functioning in older adults. The study follows a representative sample of older adults in the Netherlands since 1992 ^1^. Data has been collected on a broad number of health dimensions. Respondents are interviewed every three years. The LASA sample consists of two cohorts. The first cohort started with 3107 respondents born between 1908 and 1937. In 2002, a new cohort was added. This cohort consisted of 1002 respondent born between 1938 and 1947.

Health services use was estimated using registry data on hospital use and LTC use. For hospital use, The Dutch Hospital Discharge Register (LMR) was used: a register of hospital admissions, providing nearly complete coverage of hospital inpatient treatments. The Register of the Administrative Office Exceptional Medical Expenses (CAK) contains records on all use of institutional LTC and formal home care in the Netherlands covered by the Exceptional Medical Expenses Act (AWBZ). Data from the LASA survey were linked with these registries at Statistics Netherlands. Linkage of data was available for the years 1995–2007 for the LMR and for the years 2004–2007 for the LTC data. Costs were calculated using the Dutch Costs of Illness Study [45, 46].

Table 2 describes the estimation sample resulting from linking the LASA survey to the health care data. Hospital costs are observed ever year, while health data is only observed once every three years or in case of death. The information on annual hospital costs in the years for which no survey data is available is included in the estimation of the model. For the years without health survey data *g*_*m*_(*c*_*i,t*_,*y*_*i,t*_|*x*_*i,t*_) in Eq. (1) is replaced by *f*(*c*_*i,t*_|*x*_*i,t*_,*η*_*i,t*_=*m*) as explained by [31].

**Table 2:** Number of observations per year in the estimation sample. The sample consists of administrative hospital data (available each year) and survey data on health indicators (available once every three years)

Year	LASA wave	No. Obs	With health*
1995	C	3414	547
1996	C	3437	1626
1997		3313	117
1998	D	3192	660
1999	D	3081	1351
2000		2967	99
2001	E	2873	617
2002	E/2B*	2762	1493
2003	2B	2634	500
2004		2533	99
2005	F	2428	630
2006	F	2336	1346
2007		2228	117

^*^Observations for which reported health indicators are available (reported in LASA or deceased in that particular year)

^*^2B refers to the new LASA cohort introduced in 2002. In wave F the two cohorts were combined
